# Differential adeno-associated virus mediated gene transfer to sensory neurons following intrathecal delivery by direct lumbar puncture

**DOI:** 10.1186/1744-8069-6-31

**Published:** 2010-05-28

**Authors:** Lucy Vulchanova, Daniel J Schuster, Lalitha R Belur, Maureen S Riedl, Kelly M Podetz-Pedersen, Kelley F Kitto, George L Wilcox, R Scott McIvor, Carolyn A Fairbanks

**Affiliations:** 1 Departments of Veterinary and Biomedical Sciences, University of Minnesota, Commonwealth Avenue, Saint Paul, MN 55108, USA; 2 Departments of Neuroscience, University of Minnesota, Church Street, Minneapolis, MN 55455, USA; 3 Departments of Genetics Cell Biology and Development, University of Minnesota, Church Street, Minneapolis, MN 55455, USA; 4 Departments of Pharmaceutics, University of Minnesota, Harvard Street, Minneapolis, MN 55455, USA; 5 Departments of Pharmacology, University of Minnesota, Church Street, Minneapolis, MN 55455, USA; 6 Departments of Dermatology, University of Minnesota, Delaware Street, Minneapolis, MN 55455, USA

## Abstract

**Background:**

Neuronal transduction by adeno-associated viral (AAV) vectors has been demonstrated in cortex, brainstem, cerebellum, and sensory ganglia. Intrathecal delivery of AAV serotypes that transduce neurons in dorsal root ganglia (DRG) and spinal cord offers substantial opportunities to 1) further study mechanisms underlying chronic pain, and 2) develop novel gene-based therapies for the treatment and management of chronic pain using a non-invasive delivery route with established safety margins. In this study we have compared expression patterns of AAV serotype 5 (AAV5)- and AAV serotype 8 (AAV8)-mediated gene transfer to sensory neurons following intrathecal delivery by direct lumbar puncture.

**Results:**

Intravenous mannitol pre-treatment significantly enhanced transduction of primary sensory neurons after direct lumbar puncture injection of AAV5 (rAAV5-GFP) or AAV8 (rAAV8-GFP) carrying the green fluorescent protein (GFP) gene. The presence of GFP in DRG neurons was consistent with the following evidence for primary afferent origin of the majority of GFP-positive fibers in spinal cord: 1) GFP-positive axons were evident in both dorsal roots and dorsal columns; and 2) dorsal rhizotomy, which severs the primary afferent input to spinal cord, abolished the majority of GFP labeling in dorsal horn. We found that both rAAV5-GFP and rAAV8-GFP appear to preferentially target large-diameter DRG neurons, while excluding the isolectin-B4 (IB4) -binding population of small diameter neurons. In addition, a larger proportion of CGRP-positive cells was transduced by rAAV5-GFP, compared to rAAV8-GFP.

**Conclusions:**

The present study demonstrates the feasibility of minimally invasive gene transfer to sensory neurons using direct lumbar puncture and provides evidence for differential targeting of subtypes of DRG neurons by AAV vectors.

## Introduction

Pain signals originate at the peripheral terminals of sensory neurons and via their central processes are transmitted to second order neurons in the dorsal horn of spinal cord. Gene therapy targeted to the spinal cord or sensory neurons could, after a single administration, provide long-term pain relief with reduced adverse effects relative to current standards of practice in chronic pain management. Additionally, gene transfer directed to specific neuronal subtypes within dorsal root ganglia (DRG) could greatly empower ongoing basic research focused upon delineating functional differences between distinct nociceptive neuronal populations. With respect to the panel of viral vectors available for gene transfer, AAV has the advantage of conferring stable, long-term gene expression in the absence of an inflammatory response [1]. Further, AAV vectors are able to transduce non-dividing cells (such as neurons), and can accommodate gene expression cassettes of up to 4.3 kB, which is well within the range of many genes potentially useful for pain control [2]. AAV serotype 2 (AAV2) has been the most widely studied serotype. However, recent studies of AAV serotypes 5 and 8 (AAV5 and AAV8) have demonstrated significantly increased gene transfer efficiency, and wider distribution of transduced cells in the CNS when compared with AAV2 [3,4]. In addition, self-complementary AAV8-mediated transduction of DRG neurons has been recently reported [5].

Viral vector-mediated gene transfer to DRG neurons, spinal cord neurons, glia, and pia mater has been achieved in animal models using direct tissue injection methods (intraparenchymal or intraneural) [2,6,7] or intrathecal (i.t.) injection through an atlanto-occipital catheter [5,8-10]. The primary limitations of these delivery approaches include requisite surgery and potential for inflammation and tissue damage [11-16]. Acute intrathecal (i.t.) delivery, which is applied clinically as well as for basic neuropharmacological research [17], offers a relatively non-invasive route of administration for viral vector-mediated gene transfer. However, viral vector administration by direct lumbar puncture has not previously been reported to result in appreciable transduction of spinal cord and DRG neurons [2]. In the present study, we evaluated intravenous mannitol pretreatment as a strategy for increasing the access of viral particles delivered i.t. via direct lumbar puncture to spinal cord parenchyma and DRG. Mannitol is used to control intracranial pressure [18] and to disrupt the intercellular tight junctions between endothelial cells of the CNS microvasculature [19]. It is also used intravenously (i.v.) to facilitate chemotherapeutic drug delivery to the CNS [19]. In animal models, mannitol, given i.v. as a pretreatment, enhanced intraparenchymal diffusion following intracerebroventricular (i.c.v.) delivery of AAV constructs [20,21]. Therefore, we hypothesized that i.v. pretreatment with mannitol would similarly enhance penetration of AAV vectors to the spinal cord parenchyma and to the cell bodies of DRG neurons.

Entry of AAV particles into cells is mediated by binding to a combination of cell-surface molecules acting as receptors or co-receptors, with different serotypes binding to different combinations of surface molecules. For example, AAV2 utilizes heparan sulfate proteoglycans as receptors and a number of other cell-surface molecules, such as fibroblast growth factor receptor 1 and hepatocyte growth factor receptor, as co-receptors [22-26]. Less is known about the receptors and co-receptors of AAV5 and AAV8 [22,27,28]. Differential expression of cell-surface molecules required for entry of AAV vectors may lead to differential tissue tropism among AAV serotypes. DRG neurons are heterogeneous in terms of cell soma size, myelination level, response properties, and neurochemistry, raising the possibility for differential targeting of DRG neuronal subtypes by different AAV vectors. The present study provides a quantitative evaluation of the DRG neuronal subtypes expressing green fluorescent protein (GFP) after transduction by both rAAV5-GFP and rAAV8-GFP, based on cell soma size and labeling for the neurochemical markers calcitonin gene-related peptide (CGRP), isolectin-B4 (IB4), and substance P (SP).

## Results

**Figure 1:**
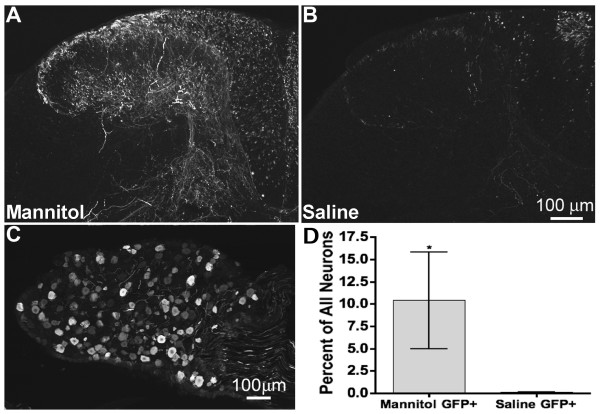
**Mannitol enhances GFP expression in mouse spinal cord and DRG**. Sections are from mice perfused six weeks after i.t. injection of rAAV5-GFP with or without intravenous mannitol pretreatment. **A) **GFP immunoreactivity (-ir) in the spinal cord of a mannitol-pretreated mouse. **B) **GFP-ir in spinal cord of a saline-pretreated mouse. **C) **GFP-ir in L4 DRG of a mannitol-pretreated mouse. **D) **Mannitol pretreatment significantly enhances transduction of lumbar DRG neurons when compared to saline pretreatment; Mann-Whitney, U_05(1),3,3 _= 9, p = 0.05.

rAAV5-GFP or rAAV8-GFP were injected intrathecally by lumbar puncture as described in Methods. GFP expression following rAAV5-GFP administration was substantially more abundant in spinal cords from mannitol-pretreated compared to saline-pretreated mice (Fig. 1A and 1B). GFP was also present in DRG neurons of mannitol-pretreated mice (Fig 1C). The number of GFP-positive DRG neurons was significantly higher (Mann-Whitney, U_05(1),3,3 _= 9, p = 0.05) in lumbar DRG of mannitol-pretreated mice compared to that of saline-pretreated mice (Fig. 1C and 1D). Intravenous pretreatment with mannitol similarly resulted in higher levels of GFP in spinal cord and DRG following i.t. administration of rAAV8-GFP (data not shown).

**Figure 2:**
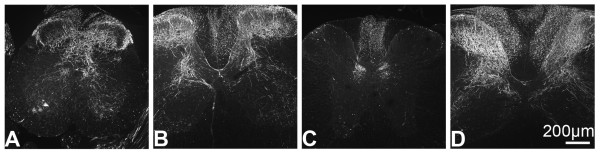
**GFP-ir in representative sections of mouse spinal cord**. **A) **Sacral, **B) **lumbar, **C) **thoracic, and **D) **cervical spinal cord sections from mice perfused six weeks after i.v. mannitol pretreatment and i.t. rAAV5-GFP injection.

**Figure 3:**
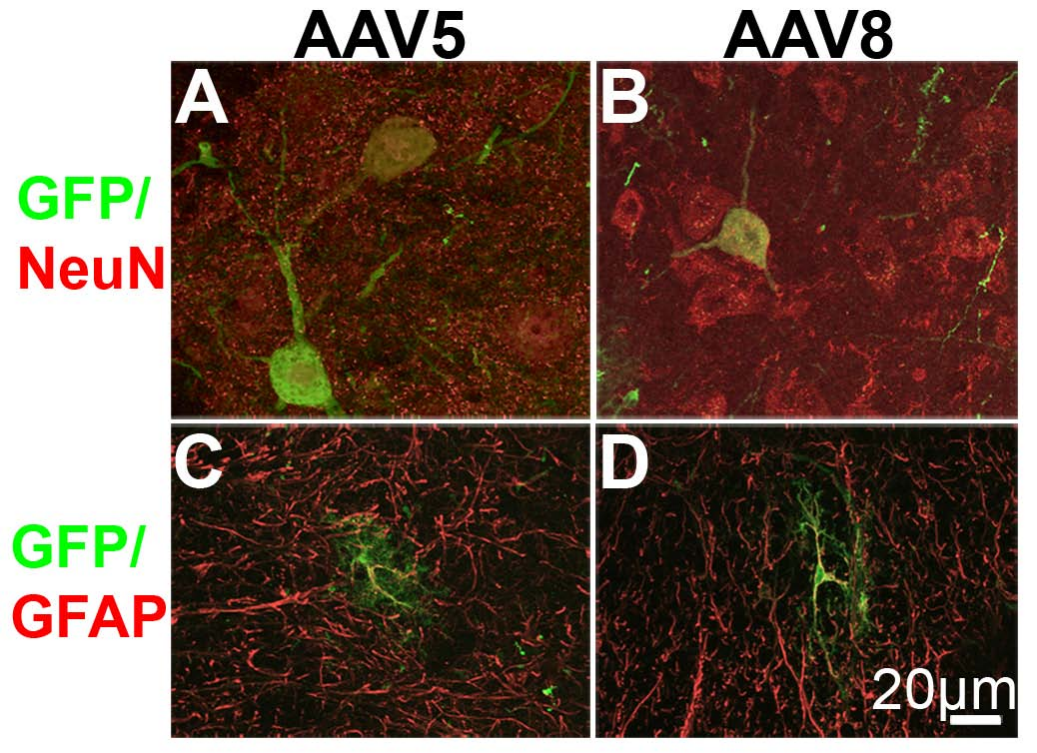
**GFP-ir in neurons and astrocytes within the ventral horn of mouse lumbar spinal cord**. GFP-ir (green) is observed in both large neurons expressing NeuN-ir (red, **A and B**) and astrocytes expressing GFAP-ir (red, **C and D)**.

GFP was observed at all levels of mouse spinal cord following treatment with rAAV5-GFP (Fig. 2) and rAAV8-GFP (not shown) with notable differences in the caudal-rostral distribution. GFP was abundant in the dorsal horn at sacral (Fig. 2A), lumbar (Fig. 2B), and cervical levels (Fig. 2D). In contrast, labeling in thoracic spinal cord was more limited and appeared to be restricted to the dorsal columns and Clarke's Columns (Fig. 2C). At all levels of spinal cord, GFP appeared to be associated predominantly with nerve fibers. However, it was generally absent from the substantia gelatinosa (lamina II) of dorsal horn. Labeling of a small number of spinal cord neurons was most evident at lumbosacral levels where GFP neurons were seen in both dorsal (not shown) and ventral horn (Fig. 3A and 3B). In addition, double labeling with GFAP identified a small number of GFP-positive astrocytes (Fig. 3C and 3D). Reducing the injection volume (and consequently the viral load) resulted in predominantly lumbosacral localization of GFP-ir (data not shown).

**Figure 4:**
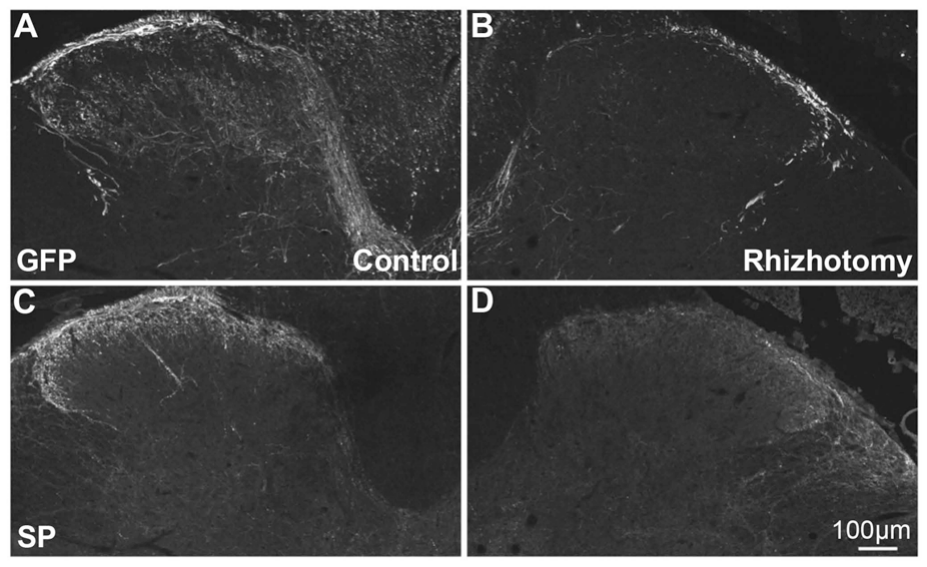
**Dorsal rhizotomy reduces GFP and SP labeling in spinal cord**. GFP- (**A **and **B**) and substance P-ir (**C **and **D**) in rat lumbar spinal cord. Unilateral dorsal rhizotomy was performed 6 weeks after i.v. mannitol pretreatment and i.t. rAAV5-GFP injection. GFP-ir is reduced ipsilaterally to dorsal rhizotomy **(A and B)**. Reduction in SP-ir in the same section (**C and D**) demonstrates the efficacy of the surgical procedure.

To determine whether the GFP observed in fibers of the dorsal horn originated from primary afferent neurons whose cell bodies are found in DRG, dorsal rhizotomies were performed in rAAV5-GFP injected rats (6 weeks post-injection) to sever input from the DRG. The pattern of labeling in rat was consistent with that of mouse at all spinal cord levels. GFP was notably reduced in the dorsal horn ipsilateral to the severed dorsal roots in contrast to abundant GFP labeling on the contralateral, unoperated side (Fig. 4A and 4C). Double-labeling with antiserum directed against substance P (SP) showed that SP-ir was also reduced in the ipsilateral dorsal horn of rhizotomized rats (Fig. 4B and 4D), confirming the disruption of afferent input. Further evidence suggesting GFP localization in the central processes of primary afferent neurons was the observation of labeling in dorsal roots as well as in the dorsal columns.

**Figure 5:**
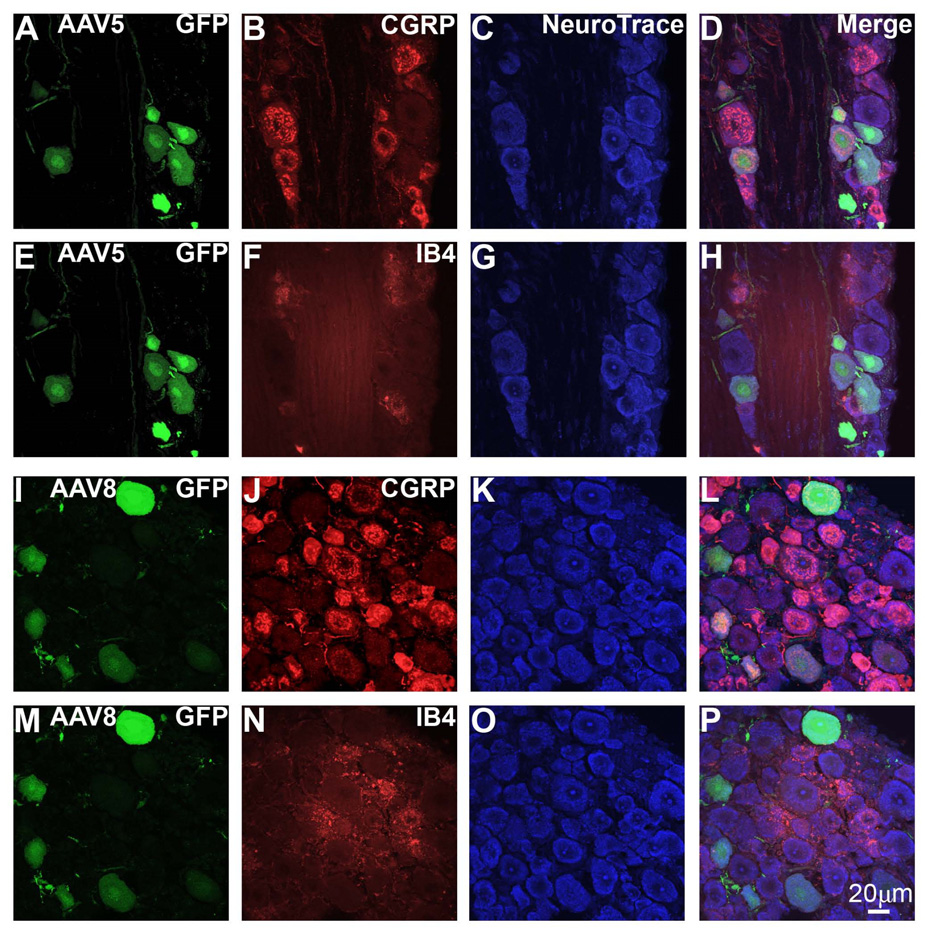
**GFP expression in DRG**. GFP-ir after i.v. mannitol pretreatment and i.t. injection of either rAAV5-GFP (**A-H)**, or rAAV8-GFP (**I-P**) in mouse L5 DRG. GFP-ir (green) is found in cells expressing CGRP-ir (red) (**A-D and I-L) **but not in cells that bind IB4 (red) (**E-H and M-P)**

**Figure 6:**
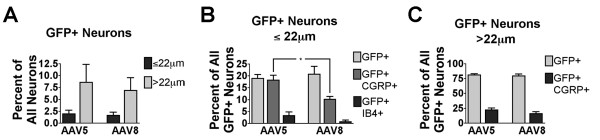
**Quantitative image analysis of GFP-expressing DRG neurons**. **A) **A similar number of neurons smaller than 22 μm (≤22 μm), or larger than 22 μm (>22 μm) expressed GFP after both rAAV5-GFP and rAAV8-GFP injection. Both vectors preferentially targeted large neurons. **B) **Injection of rAAV5-GFP resulted in more GFP expression in CGRP-positive neurons smaller than 22 μm than was seen with injection of rAAV8-GFP; t-test, t_.05,2,4 _= 3.421, p = 0.0268. Injection of either vector did not result in GFP expression in a substantial portion of the IB4-binding population. **C) **Approximately 80% of the DRG neurons expressing GFP were larger than 22 μm, regardless of whether rAAV5-GFP or rAAV8-GFP was injected. Approximately 20% of these GFP-expressing neurons were also CGRP positive.

**Figure 7:**
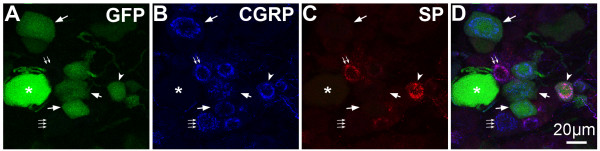
**Colocalization of GFP-ir with SP- and CGRP-ir in DRG Neurons**. GFP colocalization with CGRP and SP after i.t. delivery of AAV5-GFP in mice. **A) **GFP fluorescence. **B) **CGRP-ir. **C) **SP-ir. **D) **Merged image. Of 10 GFP-positive neurons in this image, 6 show CGRP-ir (indicated by single arrows), and of those 6, 1 also displays SP-ir (indicated by arrowhead).

In mouse DRG, GFP was seen in neuronal cell bodies and fibers after both rAAV5-GFP (as shown in Fig 1C) and rAAV8-GFP treatment (data not shown). However, in rAAV8-GFP treated DRG, GFP labeling was also observed in non-neuronal cells, which did not co-label with markers of glial cells (data not shown). Quantitative image analysis of DRG from mice injected with rAAV5-GFP (Fig. 5A-H) or rAAV8-GFP (Fig. 5I-P) was performed in DRG sections labeled with anti-CGRP and IB4 and counterstained with NeuroTrace (Fig. 5). The proportions of CGRP-positive and IB4-positive neurons determined in these experiments (AAV5: CGRP, 37.0 +/- 1.6%, IB4, 23.4 +/- 1.7%; AAV8: CGRP, 28.6 +/- 0.5%; IB4, 24.4 +/- 2.2%) were consistent with previous reports [29-31]. Notably, we found that approximately 30% of neurons with diameter less than 22 μm were not labeled by either anti-CGRP or IB4 (AAV5: 31.3 +/- 1.8%; AAV8: 35.6 +/- 2.9%). GFP-ir was observed predominantly in neurons with diameter greater than 22 μm (Fig. 6A). Of the GFP-positive neurons with diameter less than 22 μm, a larger proportion was CGRP-positive following rAAV5-GFP than following rAAV8-GFP treatment: t-test, t_.05,2,4 _= 3.421, p < 0.05 (Fig. 6B). Double labeling with anti-CGRP and anti-SP showed that both peptides were present in a subset of GFP-positive neurons after rAAV5-GFP treatment (Fig. 7). The number of IB4/GFP-positive neurons after both rAAV5-GFP and rAAV8-GFP was very limited (Fig. 5H and 5P, and Fig. 6B).

## Discussion

The present study demonstrates the feasibility of gene transfer to sensory neurons using direct lumbar puncture. As a delivery method to the intrathecal space, direct lumbar puncture is both minimally invasive and minimally damaging to the spinal cord parenchyma since it does not require surgery or catheter maintenance. The utility of gene transfer to sensory neurons for furthering studies of pain pathways as well as potentially for pain management at the spinal level would be increased by the development of viral vectors that selectively target subtypes of sensory neurons. To that end, our results provide evidence for differential targeting of DRG neurons by AAV vectors.

Intrathecal delivery using the atlanto-occipital catheterization approach [8] has successfully achieved gene transfer at the level of the pia mater [9], intrinsic spinal cord neurons, and sensory neurons [5,10]. The strength of the i.t. catheterization approach has largely been its application for reliable continuous infusion or repeated injections [11]. However, given that a single AAV injection is sufficient to transduce a substantive number of neurons, optimization of a less invasive non-surgical delivery approach in animal models is warranted. Due to its hyperosmotic properties, mannitol compromises the tight junctions of the blood brain barrier and consequently has been used to enhance CNS delivery of chemotherapeutic agents [19]. Furthermore, i.v. mannitol has been used in rodents to expand distribution of AAV vectors into brain parenchyma following intracerebroventricular injection [20,21]. The present study showed that the same concentration of i.v. mannitol similarly enhanced penetration of AAV5-GFP and AAV8-GFP vectors to the spinal cord parenchyma and to the dorsal root ganglia. Although GFP labeling in spinal cord was present predominantly in nerve fibers, a small number of transduced dorsal and ventral horn neurons as well as astrocytes was consistently observed, suggesting that both viral vectors are capable of gaining access to these cells.

The distribution of GFP labeling in spinal cord and DRG suggests that AAV5 and AAV8 target sensory neurons differentially. The pattern of GFP-labeled nerve fibers in spinal cord was consistent with the subsets of DRG neurons identified as GFP-positive. At cervical, lumbar and sacral level, GFP labeled fibers were seen predominantly in deeper laminae of dorsal horn and in the dorsal columns, in agreement with the observation that both AAV5 and AAV8 preferentially transduce larger diameter DRG neurons. The pattern of GFP labeling in thoracic spinal cord was notably different from other levels (in both rat and mouse) and appeared restricted to the dorsal columns and Clarke's columns. The reason for this difference is unknown but may be related to differential targeting of proprioceptive primary afferent neurons in thoracic DRG. In contrast to AAV5 and AAV8, a recent study reported that AAV6 targets predominantly small diameter DRG neurons [10]. AAV5- or AAV8-mediated gene transfer could be useful for manipulating protein expression in large sensory neurons, which commonly signal innocuous stimuli, but may also carry pain signals in allodynic conditions [32].

GFP-labeled fibers were also present in lamina I of the spinal cord. These fibers most likely represented collaterals of large diameter neurons as well as terminations of the small subset of peptidergic neurons that was targeted by the vectors. The distribution of GFP in superficial dorsal horn from AAV5- and AAV8-treated mice was similar. However, quantitative analysis in DRG indicated that while nearly all of the rAAV5-GFP transduced neurons with diameter less than 22 μm were also CGRP-positive, rAAV8-GFP transduced a significantly smaller proportion of CGRP-positive neurons of this size. Since the population of CGRP neurons is heterogeneous, this observation may indicate differential expression of receptors for AAV5 and AAV8 within the different subsets of CGRP-positive neurons.

It is noteworthy that GFP labeling was minimal in lamina II (substantia gelatinosa, SG) at all levels of the spinal cord. Similar observations have been made in studies of AAV2-MOR expression following intraneural injection [6] and AAV8-GFP following i.t. injection by the atlanto-occipital catheterization method [5]. The reason for absence of GFP-ir in the SG is unclear. It is likely that, in our experiments, the low levels of GFP in SG are due to the limited targeting of neurons smaller than 22 μm, in particular the minimal transduction of IB4-binding neurons. Interestingly, AAV6-GFP delivery to the intrathecal space via the atlanto-occipital approach resulted in substantial transduction of IB4-positive neurons and in GFP labeling in the SG of the spinal cord [10].

The observation that neither AAV5 nor AAV8 transduced an appreciable number of IB4-positive neurons suggests that these neurons lack a surface molecule required for the entry of these vectors. While AAV5 and AAV8 may have limited application for transduction of mouse IB4-positive neurons, this limitation can be advantageous for studies targeting IB4-negative neuronal populations. In addition, the targeting of rat IB4-positive neurons in by AAV5 and AAV8 may be different from that of mouse, since there are notable species differences in the neurochemical subsets of DRG neurons [31]. The finding by Towne and colleagues [10] that AAV6 is able to transduce mouse IB4-positive neurons indicates that AAV6 is able to enter cells via different cell-surface molecules than AAV5 and AAV8, and further supports differential targeting selectivity of neuronal subpopulations by different AAV vectors. This evidence highlights the importance of quantitative analysis of colocalization with histochemical markers of neuronal sub-populations for accurate evaluation of the tropism of various AAV vectors.

## Conclusion

Although several groups have tried with varied success to use AAV vectors to transduce cells of the DRG or spinal cord following intraparenchymal delivery, intraneural injection, or various i.t. delivery approaches [2], none has reported abundant transduction of the DRG or of the deeper laminae of the dorsal horn without a delivery method requiring surgical intervention. We have demonstrated widespread expression of GFP in the spinal cord and select subsets of DRG neurons following i.t. administration of rAAV5-GFP or rAAV8-GFP via direct lumbar puncture in conscious animals pretreated with i.v. mannitol. The distinct patterns of transduction observed with AAV5 and AAV8 in the present study and with AAV6 in the study by Towne and colleagues [10] indicate that differential targeting of DRG sub-populations by various AAV vectors should be further investigated with the goal of capitalizing upon such differences for both research and therapeutic practices. Differential AAV-mediated gene transfer to spinal cord and DRG neurons may present multiple opportunities for genetic manipulation of proteins that drive the processes involved in pain sensation or inhibitory control of pain.

## Methods

### AAV Vector and Packaging

AAV vector TRUF11, containing a CAGS-regulated GFP sequence, has been previously described [1]. Packaging using AAV5 or AAV8 serotype capsid was carried out at the University of Florida Vector Core Lab of the Gene Therapy Center (Gainesville, Florida) as previously described [1].

### Animal procedures

Experimental subjects were 20 to 25 g adult male C57BL/6 mice (Harlan, Madison, WI). In one set of experiments, Sprague Dawley rats (male and female), were included as the subjects for Fig. 4 because their size renders them more appropriate for dorsal rhizotomy experiments. All experiments were reviewed and approved by the Institutional Animal Care and Use Committee (IACUC) of the University of Minnesota.

### Injections

Subjects were injected via the tail vein with 25% mannitol solution (200 μL) twenty minutes prior to i.t. injection of the viral constructs. AAV constructs were delivered intrathecally by direct lumbar puncture in awake mice [17,33] or rats [34] by an experimenter (KFK) with extensive experience in this method of drug delivery. A minor modification of the protocol was required to conserve AAV vector. The needle (a 30-gauge, 0.5-inch (mouse) or 1.5 inch (rat)) was connected to a length of PE10 tubing, which was then connected to a second needle that was attached to a 50-μl Luer-hub Hamilton syringe. For both rAAV5-GFP and rAAV8-GFP, 10 μL of the construct containing 10^11 ^viral vector genomes were injected i.t. The injection was administered by gripping gently the iliac crest of the rodent and inserting the needle (bevel side up) at about a 45° angle centered approximately between the hipbones. A reflexive flick of the tail indicated puncture of the dura. Following the injection, the animals were returned to the vivarium where they remained for six weeks, until the time of transcardial perfusion, fixation, and extraction of fixed spinal cord and DRG for immunohistochemical analysis.

### Dorsal Rhizotomy

In rats, i.v. mannitol pretreatment and i.t. rAAV5-GFP injection was followed 6 weeks later by unilateral dorsal rhizotomy. The rats were approximately 75 g at time of the injections and approximately 150-175 g at time of rhizhotomy and perfusion. Rats were anaesthetized with 75 mg/ml ketamine, 5 mg/kg xylazine and 1 mg/kg acepromazine injected intramuscularly. After deep, surgical anesthesia had been attained, an incision was made through the skin overlying T10-L7 of the spinal cord. The dorsal roots were exposed and severed close to the spinal column. The incision was closed with surgical staples and the animals allowed to survive for 5 days before perfusion fixation.

### Immunohistochemistry

All animals were sacrificed by perfusion fixation as previously described [35]. Briefly, animals were anaesthetized with 100 mg/kg ketamine, 5 mg/kg xylazine, and 1 mg/kg acepromazine injected i.m. and perfused with a solution of calcium-free tyrodes solution (in mM:NaCl 116, KCl 5.4, MgCl_2_·6H_2_0 1.6, MgSO_4_·7H_2_O 0.4, NaH_2_PO_4 _1.4, glucose 5.6, and NaHCO_3 _26) followed by fixative (4% paraformaldehyde and 0.2% picric acid in 0.1 M phophate buffer, pH 6.9) followed by 10% sucrose in PBS. Spinal cord and DRG were removed and incubated in 10% sucrose overnight at 4°C. Sections were cut at 14 μm thickness and thaw mounted onto gel-coated slides. Tissue sections were incubated for 1 hour at room temperature in diluent (PBS containing 0.3% Triton, 1% BSA, 1% normal donkey serum) and then incubated overnight at 4°C in primary antisera diluted in the same diluent. In some instances, GFP fluorescence was enhanced by immunostaining with antisera to GFP. Primary antibodies used were: rabbit anti-GFP, 1:500 (Invitrogen; Eugene, OR), guinea pig anti-SP; 1:500 (Neuromics; Edina, MN), rabbit anti-CGRP, 1:500 (Immunostar; Hudson, WI), mouse anti-NeuN. 1:500 (Chemicon; Temecula, CA), mouse anti-GFAP, 1:400 (Sigma; St. Louis, MO)), and/or biotinylated isolectin-B4, 10 μg/ml (Sigma; St. Louis, MO). After rinsing with PBS, sections were incubated one hour at room temperature with appropriate combinations of Cy2-, Cy3-, Cy5- (1:300), and AMCA- (1:400) conjugated secondary antisera (Jackson ImmunoResearch, West Grove, CA). Sections were rinsed again in PBS and coverslipped using glycerol and PBS containing 0.1% p-phenylenediamine (Sigma). DRG sections were also incubated for 20 minutes at room temperature with NeuroTrace^® ^Red (Invitrogen; Eugene, OR). Images were collected using confocal microscopy (Biorad MRC1024, or Olympus Fluoview1000), analyzed using Image J (NIH), and processed in Adobe Photoshop.

### Cell Counting

Three sections from each DRG were selected such that they were equally spaced and at least 70 μm apart. These sections were labeled with anti-CGRP, biotinylated-IB4, and NeuroTrace^® ^Red for analysis of co-localization and cell size. Seven non-overlapping images were taken across these three sections and analyzed using Image J to measure cell body size and fluorescence intensity. Neurons were identified and outlined based on their Nissl-like NeuroTrace^® ^labeling. Only cells with a visible nucleus were counted. The cutoff of 22 μm for categorization of neurons by size was chosen based on the width of the largest peak in size histograms of DRG neurons. For CGRP fluorescence intensity, a method adapted from that of Fang and colleagues [36] was used. The average intensity of the brightest 10% and the dimmest 10% of neurons in each image was measured and relative fluorescence intensity for each cell was defined as 100[(intensity of selected cell - average of dimmest 10%)/(average of brightest 10% - average of dimmest 10%)]. Cells with a relative fluorescence intensity of 28% or greater were considered positive, and this number consistently coincided with cells identified as positive by visual inspection. Because the number of GFP expressing cells in each section was low, an alternative method was used to determine background fluorescence of GFP. The average fluorescence of three GFP-negative cells in each image was calculated, and GFP-positive cells were defined as cells with average intensity greater than that of the 3 negative cells plus 3 standard deviations. IB4 positive cells were defined using the same method as that for GFP positive cells.

## Abbreviations

rAAV: recombinant adeno-associated virus; CGRP: calcitonin gene-related peptide; DRG: dorsal root ganglia; GFP: green fluorescent protein; GFAP: glial fibrillary acidic protein; IB4: isolectin B4; i.t: intrathecal; rAAV5: rAAV serotype 5; rAAV8: rAAV serotype 8.

## Competing interests

The authors declare that they have no competing interests.

## Authors' contributions

LV contributed to the experimental design and drafting of the manuscript. In addition, she participated in all perfusions, dissections, histochemical analyses, and imaging as well as in interpretation of results and generation of figures. She is primarily responsible for the assurance of quality of the data and the main conclusions.

DS assisted in perfusions and dissections and performed immunohistochemistry, cell quantification and imaging. He assisted in the preparation of the figures, contributed to the analysis and interpretation of the data, and edited the manuscript.

LB initiated the studies and contributed to the experimental design and edited the manuscript.

MR performed all perfusions and dissections and participated in histochemical analyses and interpretation of results. She along with LV is primarily responsible for the assurance of quality of the data and the main conclusions and edited the manuscipt.

KPP performed the IV injections.

KFK conducted all intrathecal injections of vector and is responsible for the quality assurance of this key technique.

GLW participated in the experimental design and edited the manuscript.

RSM initiated the study with GLW and CF as collaborators and edited the manuscript.

CF organized the team, contributed to the experimental design, drafted and edited the manuscript and supported the studies.

All authors read and approved the final manuscript.
